# Psychological distress related to Covid-19 in healthy public (CORPD): A statistical method for assessing the validation of scale

**DOI:** 10.1016/j.mex.2022.101645

**Published:** 2022-02-24

**Authors:** Cong Doanh Duong

**Affiliations:** Faculty of Business Management, National Economics University, Hanoi, Vietnam

**Keywords:** Anxiety and fear of covid-19, Suspicious of being inflected by covid-19, Confirmatory factor analysis

## Abstract

Using the empirically statistical method, such as Cronbach's alpha, exploratory factor analysis, and confirmatory factor analysis, to assess the validation of the scales, which reflected the psychological distress related to Covid-19. The scale of covid-19 related psychological distress in healthy public, developed by Feng et al. (2020), has been measured by two factors, including anxiety and fear of being inflected by covid-19 (AF) and suspicious of being inflected by covid-19 (SU). Common method variable was employed to confirm that common method bias was not a major issue in this study.

• This study confirmed that both anxiety and fear of being inflected by covid-19 and suspicious of being inflected by covid-19 has been validated with 6 items.
• The results of this study provided valid scales that indicated psychological distress involved in covid-19 for further studies to investigate the impacts of covid-19 pandemic on individuals’ mental health.
• The findings also served as the good references for both scholars and practitioners to inhibit the spread of covid-19.

## Graphical abstract

**Figure:**
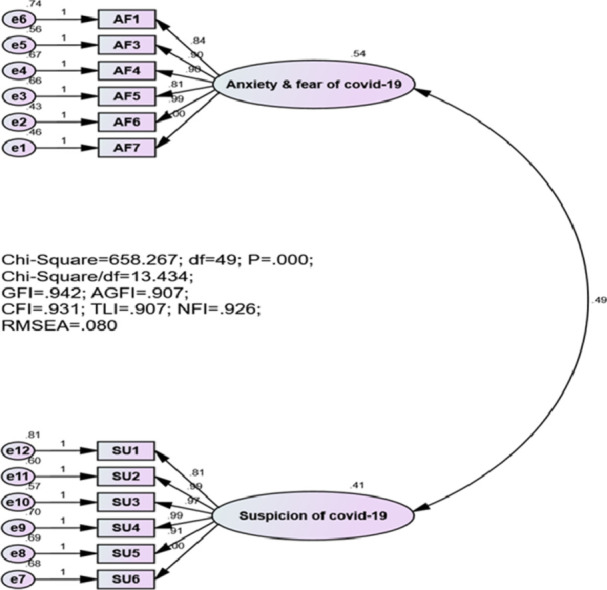

**Specifications table**

**Table:** 

Subject Area;	Psychology
More specific subject area;	Health psychology, social psychology
Method name;	Method for the assessing the validation of scale, which includes Cronbach's alpha, exploratory factor analysis (EFA) and confirmatory factor analysis (CFA)
Name and reference of original method;	Duong, C.D. (2021). The impact of fear and anxiety of covid-19 on life satisfaction: Psychological distress and sleep disturbance as mediators. *Personality and Individual Differences*, 178, 110,869. https://doi.org/10.1016/j.paid.2021.110869 Feng, L., Dong, Z., Yan, R., Wu, X., Zhang, L., Ma, J., & Zeng, Y. (2020). Psychological distress in the shadow of the covid-19 pandemic: Preliminary development of an assessment scale. Psychiatry Research, 291, 113,202. https://doi.org/10.1016/j.psychres.2020.113202
Resource availability;	https://doi.org/10.1016/j.paid.2021.110869 https://doi.org/10.1016/j.psychres.2020.113202

## Introduction

The specter of Covid-19 pandemic has been looming over the global economy. The Covid-19 has been responsible for a huge body of deaths worldwide [1]. This pandemic not only poses serious threats to our lives of those infected in influenced regions, but it also leads various degrees of negative and profound mental and psychological health problems to both infected and uninfected healthy individuals [2,3]. Psychological distress is determined as unpleasant emotional experiences that resulted from a variety of antecedents, such as fear, tension, anxiety, and psychological instability. Serious psychological issues such as depression can be derived from certain distress while infectious diseases can be seen as one of the culprits of psychological distress [4]. The link between the risk of death from viral infectious disease and psychological distress was also found in previous studies [5].

Since current outbreak, some prior studies have illustrated a fast increase of mental disorders such as psychological distress, in uninfected healthy individuals as a result of the growing risk of Covid-19 infection [2], strict quarantine measures, compulsory home quarantine and other occurrences [6,7]. It is specifically crucial to consider more exactly whether uninflected healthy individuals, such as students, are suffering the psychological distress related to covid-19. Feng et al [2]. suggested that effective and reliable assessment tool is necessary to find out these mental problems, including anxiety & fear and suspicion of covid-19. This study also developed and assessed the validity of this scale in the context of China. However, this study did not illustrate the average variance extracted (AVE) and composite reliability (CR) of each scale. Thus, the convergent validity and discriminant validity of scales is still questionable.

Moreover, Feng et al [2]. also adopted the method conjoined convenient sampling with snowball sampling, nonetheless, the sample size is rather small and mostly recruited from Yunnan province (China), so, there resulted to bias in their research results. They also call for further research to test these scales in different regions and various population. Therefore, the reliability and the validity of the scale of Psychological Distress related to Covid-19, which has been developed by Feng et al [2], was tested with the dataset from Vietnamese students in this article.

## Methodology and data

The method of quantitative analysis was conducted to analyze the data. To specific, firstly some descriptive statistics such as mean, standard deviation, skewness and kurtosis were demonstrated to show the characteristics of scales, then, the Cronbach's alpha and confirmatory factor analysis (CFA) were utilized to test the internal reliability, validity of scales, and the fit of model. Especially, this study used the scale of “psychological distress” (PD), which has been developed by Kessler et al [8], as a reference to evaluate the validity of criterion. Finally, the common method variable was used to confirm the consistent absence of biases.

Two primary constructs in this survey were measured using scales which developed by the previous study. The scale measured psychological distress related to Covid-19 (14 items), which includes anxiety & fear, suspicious of being infected by Covid-19, was adopted from Feng et al [2]. Also, because the target respondents are youths from Vietnam, therefore, the scale items were first translated into Vietnamese from the original English version. Some words have been modified to be more appropriate to the Vietnamese context. Then, the questionnaire instrument was back-translated into English to guarantee the consistence of original and translational versions. The questions were rated in a five Likert-type format from 1 (strongly disagree) to 5 (strongly agree). The survey carried out during the period from 20 September 2020 to 30 December 2020. The sample includes 1926 undergraduate students recruited from universities in Vietnam using convenience sampling technique through an online questionnaire. Particularly, the online survey delivered to students utilizing Email, Facebook and LinkedIn.

The supplementary of raw dataset in that case refers to psychology distress related to covid-19, which including anxiety & fear dimension and Suspicion dimension. *Firstly*, the dataset aims to provide the raw data, which was collected from university students, to represent their perceptions about psychological distress related to covid-19. *Secondly*, some demographic characteristics of respondents are also included in the dataset. *Finally*, the dataset is utilized to demonstrate the statistical evidence of the validity and reliability of the psychology distress related to covid-19 scales. To accomplish the research objectives, all data were processed through SPSS 24.0 and AMOS 24.0.

**Fig. 1:**
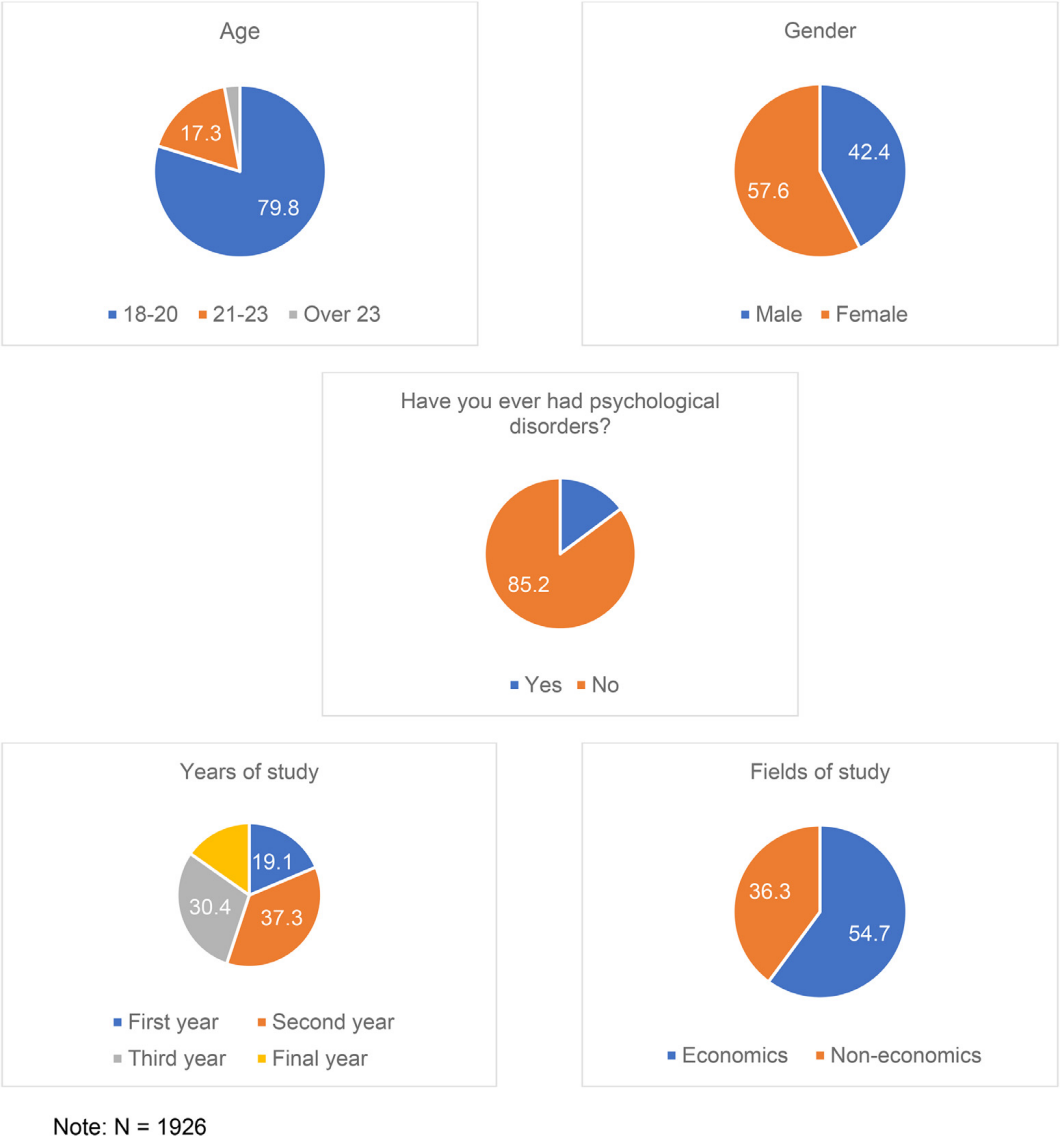
Demographic profile of respondents.

The questionnaire consisted of two major information sections: socio-demographic information and measurement constructs. Particularly, the first section included the information about respondents’ characteristics, including age (3 categories: 18–20; 21–23; over 23 years old), gender (2 categories: Male and Female), years of study (4 categories: First year; Second year; Third year; and Final year), field of study (2 categories: Economics and Non-economics), have you ever had psychological disorders? (2 categories: Yes and No). Respondents’ profiles are represented in Fig. 1.

**Fig. 2:**
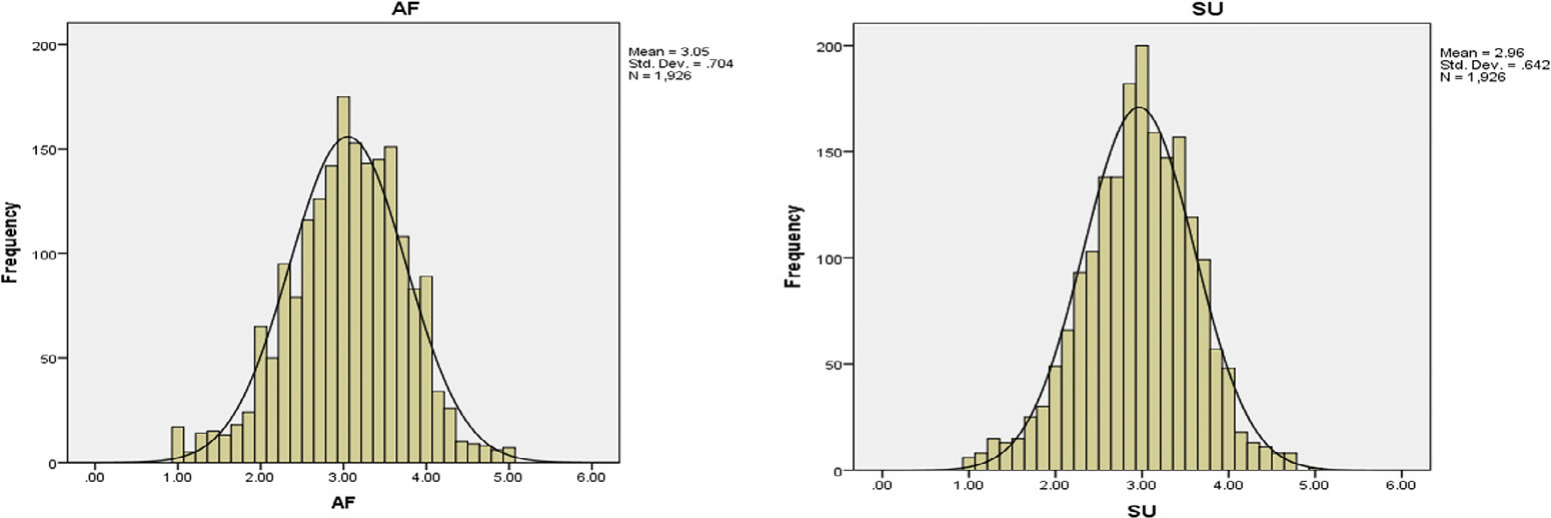
Histograms with normal curve.

The second section contained items related to psychological distress related to covid-19 and symptoms referred to mental disorders. Feng et al. suggested that the scale of psychological distress related to covid-19 in healthy public (CORPD) could be divided into two dimensions: Anxiety & fear and Suspicion being infected by Covid-19. The results of Cronbach’ alpha and the descriptive characteristics (mean, standard deviation, skewness and kurtosis) of scales was described in Table 1. All items in the scales had the values within expected levels, the skewness values are lower than 3 and the kurtosis value are less than 8 [9]. Also, all variables were also able to be acceptable when their values of Cronbach's alpha are higher than 0.63 and the Corrected Item-Total Correlation of each item is higher than 0.3 [10]. The KMO and Bartlett test of sphericity were used to determine the appropriateness of factor analysis. The result of exploratory factor analysis (KMO = 0.928, Sig. of Bartlett test of Sphericity < 0.001) demonstrated that good appropriateness to perform Confirmatory Factor Analysis (CFA) [11]. In addition, Fig. 2 illustrated the histograms with normal curve.

**Table 1:** Cronbach's alpha and descriptive characteristics of variables (*N* = 1926).

		Mean	SD	Skewness	Kurtosis	Cronbach's alpha
**AF**	**Anxiety & fear of being inflected by Covid-19 (Feng et al., 2020)**	**3.0525**	**0.7041**	**−0.281**	**0.056**	**0.823**
**AF1**	If I were infected with COVID-19, I might not be able to recovery from it	2.7762	1.0579	−0.037	−0.037	0.804
**AF2**	I'm afraid to travel to places hard-hit by COVID-19	3.5742	0.9575	−0.768	−0.768	0.822
**AF3**	When I see an increase in the number of COVID-19 patients on the news, I feel anxious	2.9849	1.0040	−0.087	−0.087	0.192
**AF4**	I think frequent hospital visits would make it easier to be infected with COVID-19	2.8645	1.0558	0.026	0.026	0.807
**AF5**	I fear to see the doctors and nurses who had worked in COVID-19 isolation wards	3.2051	1.0080	−0.437	−0.437	0.807
**AF6**	I think frequent use of air, train, bus and other public transport would make it easier to be infected with COVID-19	3.0135	0.9839	−0.161	−0.161	0.780
**AF7**	I fear to live nearby a COVID-19 isolation hospital	2.9491	1.0010	−0.085	−0.085	0.786
**SU**	**Suspicion of being inflected by Covid-19 (Feng et al., 2020)**	**2.9650**	**0.6420**	**−0.231**	**0.230**	**0.750**
**SU1**	When talking to a stranger, I would suspect that s/he might be infected with COVID-19	3.0148	3.5774	−0.653	−0.130	0.732
**SU2**	When I see someone sneeze, I suspect s/he might be infected with COVID-19	3.0049	3.0083	−0.143	−0.582	0.705
**SU3**	When I notice someone running a fever, I suspect s/he might be infected with COVID-19	3.1605	2.6147	0.306	−0.445	0.719
**SU4**	When I see someone vomiting, I suspect s/he might be infected with COVID-19	2.8914	3.0561	−0.207	−0.715	0.700
**SU5**	When I see someone coughing, I suspect s/he might be infected with COVID-19	3.0519	2.6350	0.246	−0.618	0.711
**SU6**	When I see someone without a mask, I suspect s/he might be infected with COVID-19	2.8296	2.7051	0.183	−0.645	0.696
**SU7**	I suspect there were novel coronavirus in the air when there were people around	2.9679	3.1584	−0.418	−0.468	0.768

Confirmatory factor analysis (CFA) was also employed to test the reliability, convergent and discriminant validity of each scale. However, the fit indices of the initial measurement model were not within the recommended level: Chi-Square (76) = 1363; Chi-Square/df = 17.940; GFI = 0.889; CFI = 0.867; TLI = 0.840; RMSEA = 0.094 [12,13]. The factor loadings of AF2 (0.448) and SU7 (0.226) were lower than 0.45 [14]. Thus, AF2 and SU7 has been dropped from the scales.

**Fig. 3:**
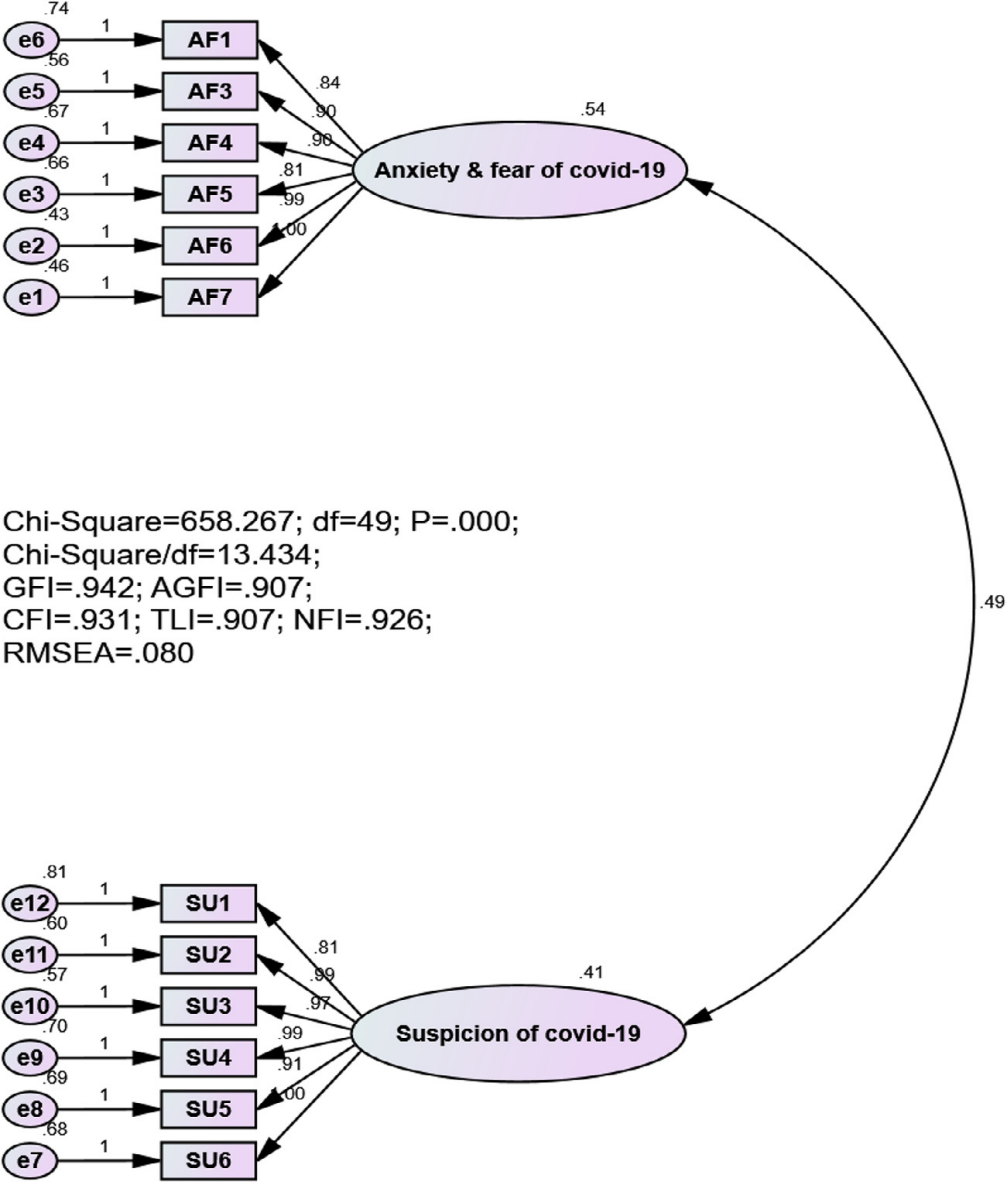
Measurement model.

After extracting unsatisfactory items, the final measurement model showed a great fitness (see Fig. 3) when the indices were within expected levels: Chi-Square (49) = 658.267; Chi-Square/df = 13.434; GFI = 0.942; CFI = 0.931; TLI = 0.907; RMSEA = 0.080 [12] Table 2. also indicated that AVE of scales are lower than 0.5. However, Fornell & Larcker [15] suggested that if AVE is less than 0.5 whereas composite reliability is higher than 0.6, the convergent validity of the construct is still adequate.

**Table 2:** Standardized regression weights of items.

Construct	Items	Estimates	Composite Reliability (CR)	Average Variance Extracted (AVE)
Anxiety & fear of being inflected by Covid-19 (AF)	AF1	0.584	0.823	0.438
	AF3	0.664		
	AF4	0.629		
	AF5	0.595		
	AF6	0.795		
	AF7	0.737		
Suspicion of being inflected by Covid-19 (SU)	SU1	0.499	0.766	0.355
	SU2	0.634		
	SU3	0.638		
	SU4	0.604		
	SU5	0.574		
	SU6	0.614		

*Note: N* = 1926.

In the criterion validity test, Pearson correlation analysis has been also employed to test the association between the total score of CORPD and the total score of PD (the reference). Our analysis result illustrated that there was a strong positive correlated between PD and CORPD (*β* = 0.356; *p*-value < 0.001). Consequently, the scale of CORD had demonstrated a very good criterion validity (see Table 3).

**Table 3:** The correlation between CORPD and PD.

Coefficients^a^						
Model		Unstandardized Coefficients		Standardized Coefficients	t	Sig.
		β	Std. Error	Beta		
1	(Constant)	1.595	.082		19.483	.000
	CORPD	.356	.027	.287	13.154	.000

a Dependent Variable: PD.

Finally, the common method variable was used to confirm that common method bias was not a major issue in this study. Firstly, Harman's one-factor test with unrotated factor solutions was employed. Result revealed that the total variance extracted by single factor for sample was 45.326%, which was lower than the recommended threshold of 50%, therefore, there was no issues with common method bias in this research [16]. Secondly, Harman's one-factor was used to carry out the confirmatory factor analysis, the result also illustrated the poor data fit: Chi-Square (54) = 981.772; Chi-Square/df = 18.181; GFI = 0.832; CFI = 0.895; TLI = 0.872; RMSEA = 0.094. Finally, the common latent variable test was performed. Then, the standardized regression weights of all items for two model have been compared, results demonstrated that there were no significant differences between standardized regression weights of items (Δ < 0.2). Thus, there was not existing common method variance with this data [15].

Subgroup analysis has been conducted via one-way ANOVA. Results reported that there were statistically significances in subgroups of different gender, fields of study, and mental health (*P*-value < 0.05) (see Table 4). There were no statistically significant differences in subgroups of age and years of study (*P*-value > 0.05).

**Table 4:** Scores of the CORPD in populations with different demographic characteristics (M ± SD).

Social-demographic information		N	CORPD scores	*P*-value
Gender	Male	817	2.9062 ± 0.71789	0.018
	Female	1109	2.9808 ± 0.65522	
Age	From 18 to 20 years old	1537	2.9372 ± 0.69159	0.237
	From 21 to 23 years old	334	2.9863 ± 0.66120	
	Over 23 years old	55	3.0591 ± 0.56535	
Fields of study	Economics and business management	1054	2.9860 ± 0.67486	0.009
	Engineering and other	872	2.9046 ± 0.69119	
Years of study	First year	367	2.8953 ± 0.65908	0.358
	Second year	718	2.9674 ± 0.68664	
	Third year	586	2.9253 ± 0.70104	
	Final year	255	3.0301 ± 0.66108	
Have you been diagnosed with a mental health problem?	Yes	286	2.8680 ± 0.70184	0.029
	No	1640	2.9633 ± 0.67927	

*Note: N* = 1926, M: Mean, SD: Standard Deviation.

## Conclusions

This study employed Cronbach's alpha, exploratory factor analysis and measurement model to assess the validation of the scales, which reflected the psychological distress related to Covid-19, and measured by two factors, including anxiety and fear of being inflected by covid-19 (AF) and suspicious of being inflected by covid-19 (SU). In our empirically statistical analyses, the validation of the scales has been confirmed. In particular, the valid scale of anxiety and fear of being inflected by covid-19 (AF) contained 6 items (1-If I were inflected with covid-19, I might not be able to recovery from it; 2-When I see an increase in the number of covid-19 patients on the news, I feel anxious; 3-I think frequent hospital visits would make it easier to be infected with Covid-19; 4- I fear to see the doctors and nurses who had worked in Covid-19 isolation wards; 5- I think frequent use of air, train, bus and other public transport would make it easier to be infected with covid-19; 6- I fear to live nearby a covid-19 isolation hospital) while the valid scale of suspicious of being inflected by covid-19 also consisted of 6 items (1- When talking to a stranger, I would suspect that s/he might be infected with covid-19; 2- When I see someone sneeze, I suspect s/he might be infected with covid-19; 3- When I notice someone running a fever, I suspect s/he might be infected with covid-19;4- When I see someone vomiting, I suspect s/he might be infected with covid-19;5- When I see someone coughing, I suspect s/he might be infected with covid-19;6- When I see someone without a mask, I suspect s/he might be infected with covid-19). The study also showed the absence of common method bias. However, there are a difference in the final validated scale of COPRD of this study, compared to the final scale validated by Feng et al [2]. This difference can be driven from the different research context between Vietnam and China. At the period of this conducted study, Vietnam controlled the spread of Covid-19 pandemic effectively and impressed international communities with restricted resources [3]. Indeed, Vietnam shares a long land border, has a huge quantity of trade with China and has the large number of illegal Chinese immigrants as well as a big volume of Chinese visitors, it has announced only 35 deaths, 1395 infected cases and 1238 recovered patients since the first imported case from Wuhan was recorded on 22 January [18]. In China, the central government carried out strong measures to control the imported cases and managed the domestic cases [2], but the inflected cases and deaths in China was much higher than Vietnam.

This result of this study provided valid scales for further studies to explore the effects of covid-19 pandemic on individuals’ mental health. Also, this result of this study served as the useful references for policymakers, practitioners, and administrators to restrict the spread of covid-19 and protect youths from mental illness [[16], [17]], which resulted from strict social distance measures of the government.

## Declaration of Competing Interest

The authors declare the following financial interests/personal relationships which may be considered as potential competing interests.

## Appendix Supplementary materials

Image, application 1

Image, application 2
