# Supplementary material for: Psychological distress related to Covid-19 in healthy public (CORPD): A statistical method for assessing the validation of scale
Source: MethodsX. 2022 Feb 24;9:101645. doi: 10.1016/j.mex.2022.101645 (PMC8867959; doi:10.1016/j.mex.2022.101645)
Supplement: Supplementary file 2 [file mmc2.docx]

**QUESTIONNAIRE**

***Dear Students,***

*We are undertaking a research project on the effect of psychological distress related to Covid-19. Please do us a favor by filling of this questionnaire. This questionnaire will be use for academic purpose only and will be analyzed and interpreted accounting to the principle of anonymity.*

*Thank you so much for your support!*

1. **PSYCHOLOGICAL DISTRESS RELATED TO COVID-19 PANDEMIC**

*Please answer the following questions with five-Likert scale:*

*1= Totally disagree*

*2= Disagree*

*3= Neutral*

*4= Agree*

*5 = Totally agree*

| The item poll of scale of COvid-19 related to psychological distress in healthy public (CORPD) | | | | | |
| --- | --- | --- | --- | --- | --- |
| 1. If I were infected with COVID-19, I might not be able to recovery from it | □1 | □2 | □3 | □4 | □5 |
| 2. I'm afraid to travel to places hard-hit by COVID-19 | □1 | □2 | □3 | □4 | □5 |
| 3. When I see an increase in the number of COVID-19 patients on the news, I feel anxious | □1 | □2 | □3 | □4 | □5 |
| 4. I think frequent hospital visits would make it easier to be infected with COVID-19 | □1 | □2 | □3 | □4 | □5 |
| 5. I fear to see the doctors and nurses who had worked in COVID-19 isolation wards | □1 | □2 | □3 | □4 | □5 |
| 6. I think frequent use of air, train, bus and other public transport would make it easier to be infected with COVID-19 | □1 | □2 | □3 | □4 | □5 |
| 7. I fear to live nearby a COVID-19 isolation hospital | □1 | □2 | □3 | □4 | □5 |
| 8. When talking to a stranger, I would suspect that s/he might be infected with COVID-19 | □1 | □2 | □3 | □4 | □5 |
| 9. When I see someone sneeze, I suspect s/he might be infected with COVID-19 | □1 | □2 | □3 | □4 | □5 |
| 10. When I notice someone running a fever, I suspect s/he might be infected with COVID-19 | □1 | □2 | □3 | □4 | □5 |
| 11. When I see someone vomiting, I suspect s/he might be infected with COVID-19 | □1 | □2 | □3 | □4 | □5 |
| 12. When I see someone coughing, I suspect s/he might be infected with COVID-19 | □1 | □2 | □3 | □4 | □5 |
| 13. When I see someone without a mask, I suspect s/he might be infected with COVID-19 | □1 | □2 | □3 | □4 | □5 |
| 14. I suspect there were novel coronavirus in the air when there were people around | □1 | □2 | □3 | □4 | □5 |

1. **PSYCHOLOGICAL DISTRESS**

*Please answer the following questions with five-Likert scale:*

*1= Totally disagree*

*2= Disagree*

*3= Neutral*

*4= Agree*

*5 = Totally agree*

| **Psychological distress** | | | | | |
| --- | --- | --- | --- | --- | --- |
| I often feel tired out for no good reason | □1 | □2 | □3 | □4 | □5 |
| I often feel nervous | □1 | □2 | □3 | □4 | □5 |
| I often feel so nervous that nothing could calm me down | □1 | □2 | □3 | □4 | □5 |
| I often feel hopeless | □1 | □2 | □3 | □4 | □5 |
| I often feel restless or fidgety | □1 | □2 | □3 | □4 | □5 |
| I often feel so restless that I could not sit alone | □1 | □2 | □3 | □4 | □5 |
| I often feel depressed | □1 | □2 | □3 | □4 | □5 |
| I often feel that everything is not effort | □1 | □2 | □3 | □4 | □5 |
| I often feel so sad that nothing could cheer me up | □1 | □2 | □3 | □4 | □5 |
| I often feel worthless | □1 | □2 | □3 | □4 | □5 |

**C. PERSONAL INFORMATION**

1. Gender: □_1_ Male □_2_ Female
2. Field of studying:

□_1_ Economics

□_2_ Non-economics

1. Years of study

□_1_ First year

□_2_ Second year

□_3_ Third year

□_4_ Final year

1. Fields of economics:

□_1_ Economics

□_2_ Non-economics

1. Have you ever had psychological disorders?

□_1_ Yes

□_2_ No
